# Supplementary material for: A Putative TRAPα Protein of Microsporidia Nosema bombycis Exhibits Non-Canonical Alternative Polyadenylation in Transcripts
Source: J Fungi (Basel). 2023 Mar 25;9(4):407. doi: 10.3390/jof9040407 (PMC10142623; doi:10.3390/jof9040407)
Supplement: Supplementary file 1 [file jof-09-00407-s001.zip › jof-2272846-supplementary.pdf]

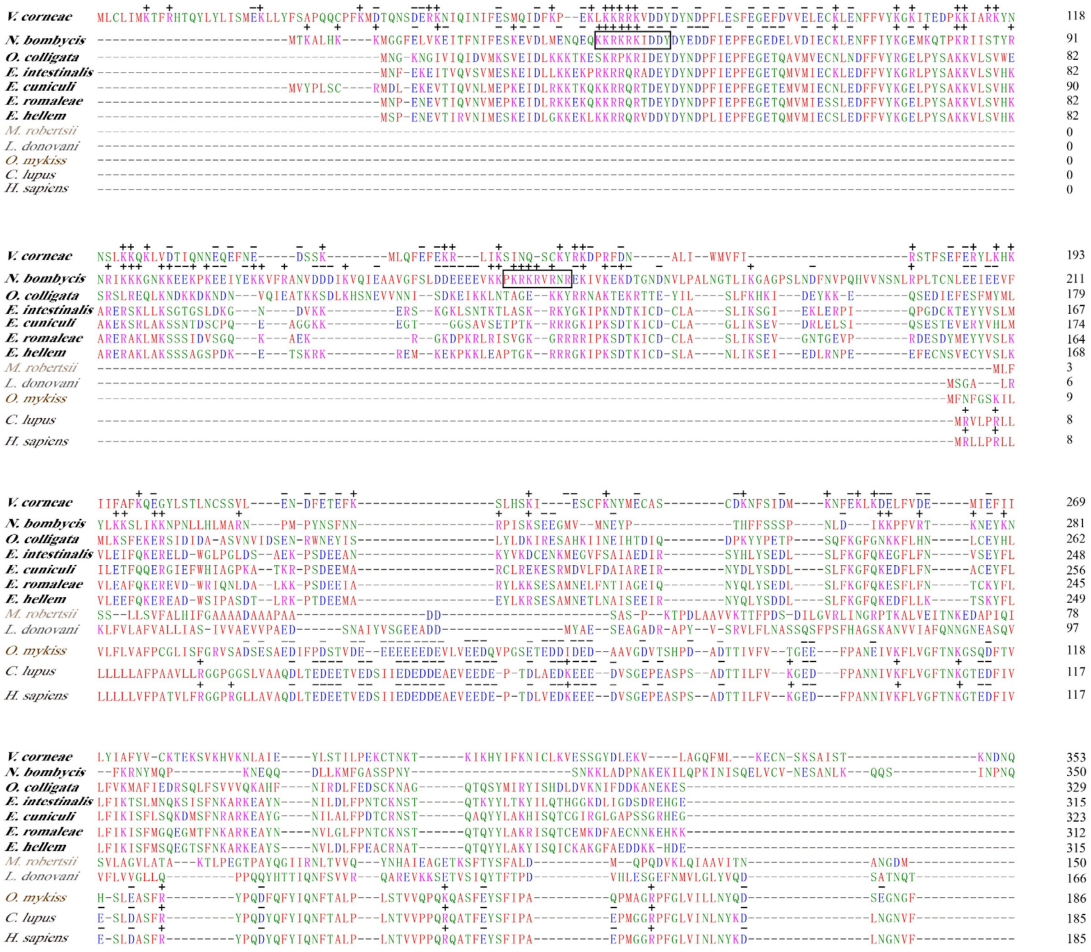

Figure S1. Multiple sequence alignment (partial) of TRAPα among fungi, pratozoa, mammal and microsporidian HTRAPα. The multiple alignment result showed that HNbTRAPα had relatively high similarity with others of microsporidia within first 100 amino acids at N terminal, but low similarity with eukaryotic TRAPα. Moreover, HNbTRAPα didn't exhibit prominent negative N-terminal. The species selected are *Nosema bombycis* (*N. bombycis*), *Vittaforma corneae* (*V. corneae*), *Ordospora colligate* (*O. colligate*), *Encephalitozoon intestinalis* (*E. intestinalis*), *Encephalitozoon cuniculi* (*E. cuniculi*), *Encephalitozoon romaleae* (*E. romaleae*), *Encephalitozoon hellem* (*E. hellem*), *Metarhizium robertsii* (*M. robertsii*), *Leishmania* donovani (*L.* donovani), *Oncorhynchus mykiss* (*O. mykiss*), *Canis lupus familiaris* (*C. lupus*) and *Homo sapiens* (*H. sapiens*). These sequences were downloaded from NCBI database (https://www.ncbi.nlm.nih.gov/) and analyzed by Clustal Omega web services (https://www.ebi.ac.uk/Tools/msa/clustalo/). Species names of different taxonomic status are identified with different colored fonts. The negative and positive charged amino acids were indicated above respectively by "＋" and "－". Two predicted importin α-dependent nuclear localization signals were marked by black rectangle.

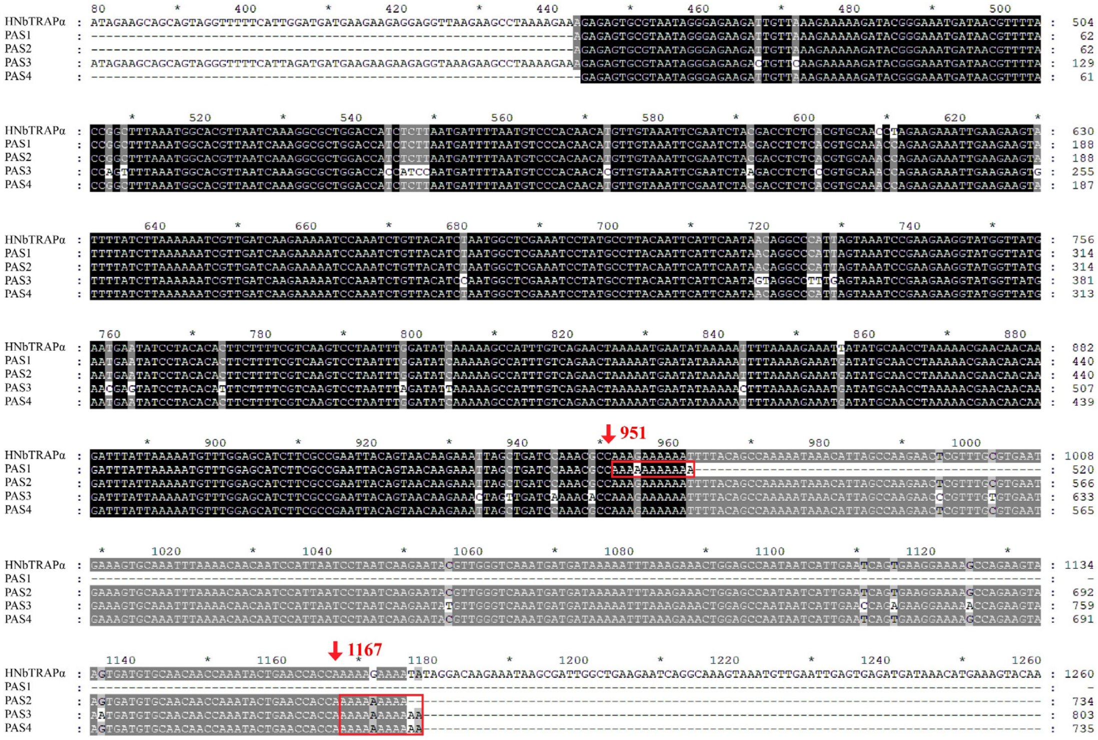

Figure S2. The align analysis of sequenced 3'-RACE products of HNbTRAP*α*. Four successfully amplified inner PCR products (named as PAS) were aligned with complete length of HNbTRAP*α*. The potential alternative split sites were indicated by red arrows that C^{951} and C^{1167} were alternative APA sites of HNbTRAP*α*. The poly(A) tails were indicated by red rectangles.
